# Effects of cotton straw and apple pomace mixed ensilage on growth performance, slaughter performance, meat quality, rumen microbiota and metabolome of Xinjiang Brown cattle

**DOI:** 10.3389/fvets.2026.1796588

**Published:** 2026-04-21

**Authors:** Ruohui Li, Shengjie Zhang, Qikai Liu, Bao Wang, Shihong Mi, Chengcheng Wang, Xiaoping Chen, Kang He, Yuan Lv, Xuelian Gao, Yuanhao Fan, Jiaru Tang, Dengke Hua, Xinfeng Wang

**Affiliations:** 1 College of Animal Science and Technology, Shihezi University, Xinjiang, China; 2 Xinjiang Production and Construction Corps 4th Division Chuangjin Agricultural Development Group Co., Ltd., Xinjiang, China; 3 Xinjiang Tianyu Biotechnology Co., Ltd., Tumushuke, China

**Keywords:** apple pomace, cotton straw, meat quality, metabolome, microbiome

## Abstract

This study aimed to investigate the effects of replacing traditional corn silage with cotton straw and apple pomace mixed ensilage feed on the growth performance, slaughter performance, meat quality, rumen microbiota and metabolome of Xinjiang Brown Cattle. Twenty 22-month-old Xinjiang Brown Cattle with similar body conditions were randomly divided into a control group (Con, corn silage) and an experimental group (Tre, cotton straw and apple pomace mixed ensilage). The experiment lasted for 180 days, during which growth performance was evaluated. At the end of the experiment, 6 cattle were randomly selected from each group for slaughter to determine slaughter performance and meat quality. Meanwhile, rumen fluid samples were collected to analyze the rumen microbial community structure using 16S rRNA gene sequencing, and rumen fluid metabolites were analyzed with untargeted metabolomics (LC–MS) technology. The results showed that there were no significant differences in growth performance (average daily gain and final weight) between the two groups. However, compared to the Con group, the carcass weight, dressing percentage and drip loss of the Tre group were significantly decreased (*p* < 0.05). Rumen fermentation results indicated that the rumen pH value of the Tre group was significantly increased (*p* < 0.05), while the acetic acid content was significantly decreased (*p* < 0.05). Microbiome analysis showed that the *α*-diversity (*Shannon* and *Chao1* indices) of rumen microbiota in the Tre group was significantly higher, and there was a significant difference in *β*-diversity (*p* < 0.05); the relative abundance of fiber-degrading bacteria such as *Fibrobacter* in the Tre group was significantly increased. LC–MS analysis revealed that the contents of beneficial metabolites such as N-Acetyl-L-methionine and Resveratrol were increased in the Tre group. In conclusion, cotton straw and apple pomace mixed ensilage feed can be used as an effective substitute for corn silage, modulating the rumen microbial community structure and altering the metabolite profile, thereby improving meat juiciness. This study provides theoretical support for the resourceful and high-value utilization of agricultural by-products such as cotton straw and fruit pomace in Xinjiang.

## 1 Introduction

Xinjiang Brown Cattle is a local characteristic breed from northern Xinjiang, China, famous for strong environmental adaptability, excellent grazing traits, and outstanding production performance (1). As the first dual-purpose (dairy and beef) cattle breed independently developed in China (2). Xinjiang Brown Cattle have dense muscle fibers, bright red color, high water-holding capacity, and good tenderness. Studies have shown that intramuscular fat (IMF) contents can reach 4.3% in this breed, considerably higher than in similar dual-purpose breeds (3, 4). Therefore, Xinjiang Brown Cattle does not only meet the market demand for high-quality meat, but also support the sustainable development of local animal husbandry.

Feed composition directly affects slaughter performance (5) and indirectly affects host energy utilization and physiological status by shaping the rumen microbiota and its metabolic activities (6, 7). Xinjiang is an arid to semi-arid region with limited natural grasslands, insufficient high-quality silage supplies, and high feeding costs (8). The region also produces large quantities of agricultural by-products from cotton and fruit industries (9, 10). Cotton straw is rich in crude fiber and contains a moderate level of crude protein, providing structural roughage and protein supplementation for ruminants (11, 12). Apple pomace contains dietary fiber and polyphenolic compounds, which can supplement both dietary fiber and functional phytochemicals (13, 14). If not properly utilized, these by-products represent wasted resources and can cause environmental pollution (15).

Currently, research on the use of cotton straw and apple pomace in ruminant production has primarily focused on the utilization of individual feed resources. Silage made from cotton straw has been widely applied in beef cattle production, and studies have shown that it can serve as a roughage substitute for partial replacement of corn silage or crop straws without significantly impairing production performance (16, 17). As a functional feed resource, research on apple pomace in ruminants has primarily focused on its effects on methane production, rumen fermentation, and antioxidant capacity (18). However, studies on the combined ensiling of cotton straw and apple pomace and their application in beef cattle production remain scarce in the literature. Cotton straw is characterized by high fiber content and insufficient fermentable carbohydrates, resulting in poor fermentation quality when ensiled alone (19). In contrast, apple pomace is rich in soluble sugars but has high moisture content, making it prone to spoilage and difficult to store independently (20). Co-ensiling these two by-products enables nutritional complementarity: the soluble sugars supplied by apple pomace promote lactic acid fermentation of cotton straw fiber, thereby enhancing silage quality, while the relatively high dry matter content of cotton straw helps adjust the moisture level of apple pomace and reduce effluent loss (21). Therefore, co-fermentation of cotton straw and apple pomace may enhance fermentation efficacy.

Therefore, this study proposes the following scientific hypothesis: mixed ensilage of cotton straw and apple pomace can modulate meat quality in Xinjiang Brown Cattle by altering the rumen microbial community and its associated metabolite profile. To test this hypothesis, Xinjiang Brown Cattle were used as the experimental model and assigned to two dietary treatments: conventional corn silage or mixed cotton straw and apple pomace ensilage. Meat quality, rumen microbiota, and metabolite profiles were comparatively evaluated between the two feeding regimes to elucidate the molecular mechanisms underlying diet-mediated regulation of meat quality and to provide a scientific basis for the efficient utilization of regional feed resources.

## 2 Materials and methods

All experimental designs and protocols were approved by the Biology Ethics Committee of Shihezi University, Xinjiang, China (Approval Number: A2025-1119) and followed the institutional guidelines for animal research.

### 2.1 Materials

The micro-ensiled feed mixture of cotton straw and apple pomace used in this experiment, including the composite bacterial inoculant, auxiliary materials, and production procedures, was provided by the Forage Comprehensive Utilization Group, College of Animal Science and Technology, Shihezi University. Experimental animals and the feed fermentation site were provided by Chuangjin Jinbian Bioengineering Co., Ltd., 62nd Regiment, Ili Kazakh Autonomous Prefecture.

### 2.2 Experimental animals and management

The animal experiment was conducted in Chuangjin Jinbian Bioengineering Co., Ltd., 62nd Regiment, Ili Kazakh Autonomous Prefecture, using a completely randomized design. Twenty 22-month-old Xinjiang Brown cattle with similar body conditions (512.42 ± 23.70 kg) and health status were selected. Based on similar body weight, animals were randomly assigned to two groups (*n* = 10 per group), and each group was housed in a separate pen. The control group (Con) received a basal diet without cotton straw; the treatment group (Tre) received a diet in which the corn silage was completely replaced by cotton straw and apple pomace mixed ensilage. The two dietary treatments were formulated to be isonitrogenous and isoenergetic. In the treatment group, the feed mixture was prepared by combining cotton straw and apple pomace at a ratio of 7:3 on a dry matter basis. The nutritional composition of cotton straw and apple pomace is shown in Table 1. The feeding adaptation period lasted 10 days, followed by a 180-day treatment period. Before the trial, the facility was fully disinfected and animals were numbered and ear-tagged. Cattle were housed in separate pens by group, with free movement within the pens. Diets were formulated with reference to the Nutritional Requirements of Xinjiang Brown Cattle. Its ingredients and nutritional composition are shown in Table 2. Each group received a total mixed ration (TMR) twice a day (9:00 and 16:00). Water was provided ad libitum, and water troughs were cleaned regularly. Daily feed offered was recorded, and residual feed was collected for three consecutive days at the beginning, middle, and end of the trial to determine dry matter intake (DMI).

**Table 1:** Nutritional composition of cotton straw and apple pomace.

Items	Cotton straw	Apple pomace
DM, g/kg FW	894	226
CP, g/kg DM	104	35.1
NDF, g/kg DM	553	454
ADF, g/kg DM	383	376
WSC, g/kg DM	13.4	129

DM, dry matter; FW, fresh weight; CP, crude protein; NDF, neutral detergent fiber; ADF, acid detergent fiber; WSC, water-soluble carbohydrates.

**Table 2:** Ingredients and nutritional composition of the control and treatment diets.

Index	Dietary treatment	
	Con	Tre
Ingredients (%, DM basis)		
Corn silage	55.30	0
Corn straw	10.10	12.90
Wheat straw	9.20	11.20
Cotton Straw and Apple Pomace mixed Ensilage	0	48.70
Wrapped baled alfalfa	7.00	8.40
Concentrate	18.40	18.80
Nutrient composition (%)		
Crude protein	13.34	13.38
Ether extract	2.98	3.28
Ash	6.31	7.23
Neutral detergent fiber	41.36	29.61
Acid detergent fiber	15.26	12.24
Calcium	0.90	0.93
Phosphorus	0.66	0.72
Metabolizable energy^3^, MJ/kg	11.34	11.53

Con: cattle fed a diet with corn silage diet; Tre: cattle fed a diet with cotton straw and apple pomace mixed ensilage; Each kilogram of premix (on a DM basis) contains: Cu 191 mg, Fe 1,200 mg, Mn 1,393 mg, Se 9 mg, vitamin A 250 KIU, vitamin E 1500IU, vitamin B1 699 mg, and niacin 1,500 mg. The levels of basic nutrients are calculated based on the measured data of each feed ingredient. ME was calculated, others were measured.

### 2.3 Determination of growth performance

During the trial, cattle were weighed every 30 days after a 17-h fasting period prior to weighing. Animals were led to the weighing platform via a biosecurity passage before morning feeding (precision 0.5 kg), and the values were used to calculate the average daily gain.

### 2.4 Determination of fermentation parameters

On the final day of the experiment, rumen fluid was collected from each beef cattle via a sterile stomach tube two hours before the morning feeding. The initial 50 mL of fluid was discarded. The collected rumen fluid was immediately filtered through four layers of gauze and its pH was measured on-site using a portable pH meter (HI-9024C, HANNA Instruments, USA). The filtered rumen fluid was then aliquoted into three 10 mL cryovials and one 50 mL centrifuge tube. The cryovials were immediately flash-frozen in a liquid nitrogen tank and subsequently transferred to a −80 °C ultra-low temperature freezer, while the centrifuge tube was stored at −20 °C for subsequent analysis of fermentation parameters.

The pH value, ammonia nitrogen, volatile fatty acid (VFA), lactic acid, and microbial protein concentrations of rumen fluid were determined. pH was measured with a portable pH meter; the ammonia nitrogen concentration was determined by salicylate-hypochlorite colorimetry; VFA concentrations were determined by gas chromatography; Lactic acid concentration was measured by a commercial kit (Nanjing Jiancheng Science & Technology Co., Ltd., Beijing, China); The concentration of microbial crude protein MCP was determined using a commercial detection kit (Beijing Solarbio Science & Technology Co., Ltd., Beijing, China).

### 2.5 Determination of slaughter performance and meat quality

At the conclusion of the experiment, six cattle with similar body weights were randomly selected from each group. Following 24 h of feed deprivation and 2 h of water withdrawal, they were transported to a commercial slaughterhouse (transport distance: 20 km; duration: approximately 30 min). Upon arrival, no injuries or signs of stress were observed in the animals. Slaughter performance was evaluated post-slaughter. The longissimus dorsi muscle between the 12th and 13th ribs on the left carcass was sampled for meat quality analysis. In the meat quality evaluation, the longissimus dorsi muscle area (eye muscle area) was determined by tracing the cross-sectional contour between the 12th and 13th ribs onto graph paper and calculating the enclosed area. After slaughter, meat samples were kept under low-light conditions for 45 min before color measurement using a calibrated colorimeter (NR10QC, 3nh, China). Five distinct points on each sample were measured, and the mean value was recorded. For shear force determination, cylindrical cores (1 × 1 cm cross-section) were extracted parallel to the muscle fibers using a coring device. The samples were vacuum-sealed in plastic bags and cooked in a water bath at 75 °C until the internal temperature reached 70 °C. After cooling to room temperature, surface moisture was gently blotted with filter paper, and samples were sheared perpendicular to the muscle fiber direction using a texture analyzer (Warner–Bratzler shear device). Ten measurements were performed per sample, and the average value was used for analysis. Drip loss and centrifugal cooking loss were determined according to the Chinese agricultural industry standard “Objective Methods for Meat Quality Evaluation” (NY/T 2798-2015), with six replicates per biological sample.

### 2.6 Microbiome sequencing and analysis

Genomic DNA was extracted from samples by a combined cetyltrimethylammonium bromide (CTAB) and sodium dodecyl sulfate (SDS) method. DNA concentration and integrity were assessed by 1% agarose gel electrophoresis, and purity was checked by A260/A280 spectrophotometry. The V3–V4 hypervariable region of the 16S rRNA gene was amplified, and each sample was introduced with a unique barcode pair during PCR. The primers used were 341F (5′-CCTAYGGGRBGCASCAG-3′) and 806R (5′-GGACTACNN GGGTATCTAAT-3′). Sequencing libraries were constructed using the NEB Next® Ultra™ DNA Library Prep Kit (NEB, USA) according to the manufacturer’s instructions and sequenced on an Illumina MiSeq or HiSeq 2500 platform.

Paired-end reads were merged using USEARCH (v11), quality-filtered, length-trimmed, and deduplicated to obtain unique sequences. Sequences were clustered into operational taxonomic units (OTUs) at 97% similarity. Taxonomic annotation was assigned by comparing representative OTU sequences to Ribosomal Database Project (RDP) 16S rRNA reference (rdp_16s_v18.fa). Alpha diversity and between-group community differences were performed using the vegan package (v2.6–4) in R (v4.4) based on the Bray–Curtis dissimilarity matrix for analysis of similarity (ANOSIM). Relative abundances at phylum and genus levels were visualized with circlize (v0.4.15) and ggplot2 (v3.4.4) packages in R.

### 2.7 Metabolome sequencing and analysis

Rumen fluid samples were thawed at 4 °C and extracted with methanol/acetonitrile (1:1, v/v) extraction solvent at a sample-to-solvent ratio of 1:2 (v/v). The mixture was vortexed for 30 s, followed by ultrasonic extraction in an ice-water bath at 5 °C for 30 min at a frequency of 40 kHz, and then incubated at −20 °C for 30 min. After centrifugation at 13,000 × g and 4 °C for 15 min, the supernatant was collected and dried using a vacuum concentrator.

For liquid chromatography-mass spectrometry (LC–MS) analysis, dried extracts were reconstituted in acetonitrile/water solution (1:1, v/v). Metabolites were separated on an ultra-high performance liquid chromatography (UHPLC, 1290 Infinity LC, Agilent) system equipped with a HILIC column and analyzed in both positive and negative electrospray ionization (ESI) modes. Quality control (QC) samples were run regularly. Mass spectrometry detection was performed on an AB Sciex TripleTOF 6600 system (AB SCIEX) with optimized ESI source parameters.

Raw MS data were converted to mzXML using ProteoWizard MSConvert. Peak extraction, alignment, and integration were performed using XCMS. Only ion features present in >50% of samples in at least one group (i.e., non-zero value ratio > 50%) were retained for subsequent analysis. Metabolites were identified by accurate mass (error < 10 ppm) and tandem mass spectrometry (MS/MS) spectra matched to an in-house standard database. After total normalization, multivariate analysis, including principal component analysis (PCA), were performed using the ROPLS package (v1.34.0) in R. Metabolites with variable importance in projection (VIP) > 1 and *p* < 0.05 were considered statistically significant.

### 2.8 Statistical analysis

All statistical analyses were performed using GraphPad Prism (version 9.0). Data are presented as means ± standard error of the mean (SEM). Prior to analysis, data normality and homogeneity of variance were assessed using the Shapiro–Wilk test and Levene’s test, respectively. Comparisons between two independent groups were conducted using an unpaired two-tailed Student’s *t*-test for data meeting the assumptions of normality and equal variance. For the analysis of microbial differences, the Wilcoxon test was employed. Correlation networks were generated using R (version 4.4.2), and heatmaps were constructed using the ComplexHeatmap package. *p* < 0.05 was considered significant, and *p* < 0.01 was considered highly significant.

## 3 Results

### 3.1 Effects of different diets on growth performance

Due to the absence of significant differences in initial body weight, there were no significant differences in average daily gain and final body weight between the Tre and Con groups (Table 3, *p* > 0.05), indicating that replacing corn silage with cotton straw and apple pomace mixed ensilage feed did not impair growth performance. Furthermore, no significant differences were observed in dry matter intake (DMI) or feed conversion ratio (FCR) between the Tre and Con groups (Table 3, *p* > 0.05).

**Table 3:** Effects of the diet with cotton straw and apple pomace mixed ensilage on growth performance of Xinjiang Brown Cattle.

Items	Con	Tre	SEM	*p*-value
Initial weight (kg)	520.50	504.33	6.841	0.256
Final weight (kg)	657.58	641.67	6.464	0.235
Average daily gain (ADG, kg/d)	0.66	0.66	0.027	0.977
DMI (kg/d)	12.37	11.61	0.464	0.440
FCR	18.75	17.59	0.703	0.439

Con group: cattle fed with corn silage diet; Tre group: cattle fed with cotton straw and apple pomace mixed ensilage feed; SEM = pooled standard error between groups; *p*-value indicated the significance of difference between the two groups, *p* < 0.05 meant significant difference, *p* < 0.01 meant extremely significant difference.

### 3.2 Effects of different diets on slaughter performance and meat quality

There were no significant differences in pH, eye muscle area, shear force, cooking loss, or muscle L*, a*, and b* values between groups (Table 4, *p* > 0.05). However, carcass weight, dressing percentage, and drip loss were significantly higher in the Con group than in the Tre group (Table 4, *p* < 0.05).

**Table 4:** Effects of the diet with cotton straw and apple pomace mixed ensilage on slaughter performance and meat quality of Xinjiang Brown Cattle.

Items	Con	Tre	SEM	*p*-value
Carcass weight (kg)	369.67	345.83	5.000	0.008
Dressing percentage (%)	56.25	53.89	0.520	0.014
pH	5.49	5.47	0.015	0.462
Eye muscle area (cm^2^)	61.81	58.83	0.829	0.069
Shear force (*N*)	34.56	33.28	1.406	0.672
Drip loss (%)	0.99	0.98	0.003	0.002
Cooking loss (%)	0.36	0.38	0.009	0.345
Meat color				
Lightness L*	34.46	34.12	0.529	0.765
Redness a*	9.57	9.95	0.281	0.534
Yellowness b*	11.05	10.67	0.351	0.607

Con and Tre groups are defined as in Table 2; *p*-value indicated the significance of difference between the two groups, *p* < 0.05 meant significant difference, *p* < 0.01 meant extremely significant difference.

### 3.3 Effects of different diets on rumen fermentation parameters

As shown in Table 5, rumen fluid pH in Tre group was significantly higher than that in Con group (*p* < 0.05), although both were within the normal range. Acetic acid and propionic acid concentration was lower in Tre group compared with Con group (Table 5, *p* < 0.01). No significant differences were found between groups for lactic acid, isobutyric acid, butyric acid, isovaleric acid, valeric acid, and MCP (Table 5, *p* > 0.05).

**Table 5:** Effects of the diet with cotton straw and apple pomace mixed ensilage on rumen fermentation parameters of Xinjiang Brown Cattle.

Items	Con	Tre	SEM	*p*-value
pH	6.03	6.86	0.178	0.011
NH_3_-N (mg/dL)	12.19	14.23	0.613	0.095
MCP (mg/mL)	2.68	2.70	0.011	0.412
Lactic acid (mmol/L)	0.32	0.41	0.014	0.320
TVFA (mmol/L)	73.89	65.45	1.669	0.004
Acetic acid (mmol/L)	48.41	42.62	1.193	0.007
Propioni acid (mmol/L)	14.24	12.75	0.364	0.034
Isobutyric acid (mmol/L)	0.58	0.50	0.032	0.266
Butyric acid (mmol/L)	7.64	6.75	0.280	0.113
Isovaleric acid (mmol/L)	1.59	1.45	0.090	0.457
Valeric acid (mmol/L)	1.44	1.38	0.038	0.480
Acetate/propionate	3.40	3.35	0.041	0.518

Con and Tre groups are defined as in Table 2. *p*-value indicated the significance of difference between the two groups, *p* < 0.05 meant significant difference, *p* < 0.01 meant extremely significant difference.

### 3.4 Effects of different diets on rumen microbiota of Xinjiang Brown cattle

**Figure 1:**
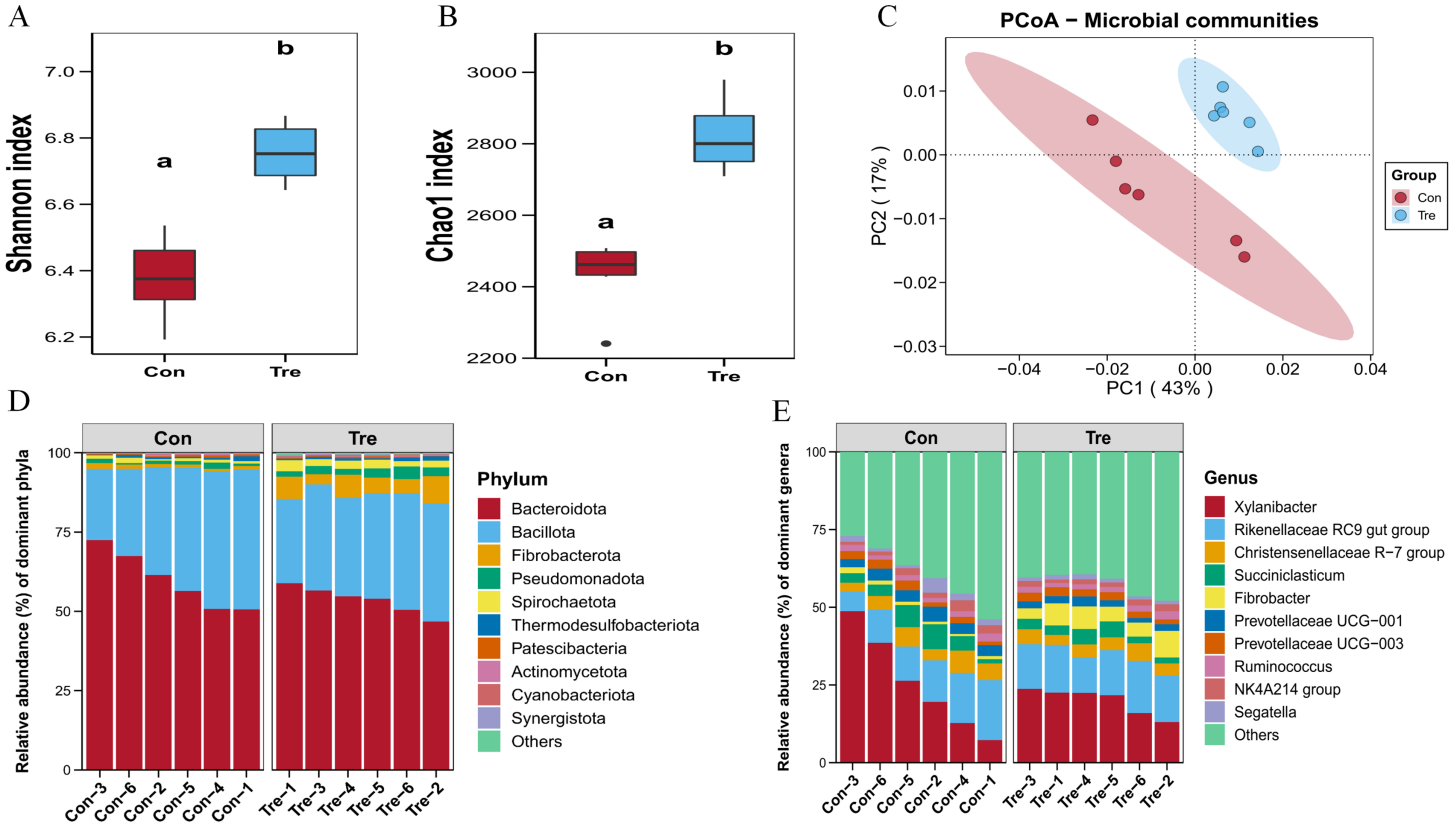
Effects of the diet with cotton straw and apple pomace mixed ensilage on rumen microbial diversity and composition of Xinjiang Brown Cattle. **(A)** Comparison of Shannon index. **(B)** Comparison of Chao1 index. **(C)** PCoA analysis. **(D)** Rumen flora composition at the phylum level. **(E)** Flora composition at the genus level. a-b means within a row with different subscripts differ when *p* < 0.05.

Alpha diversity metrics showed that both *Shannon* and *Chao1* indices were significantly higher in the Tre group than in the Con group (Figures 1A,B, *p* < 0.05), indicating greater microbial diversity and richness with the Tre diet. In addition, PCoA analysis revealed significant differences in *β*-diversity between groups (Figure 1C, *p* < 0.05). At the phylum level, Bacillota and Bacteroidota (Bacteroidetes) dominated in both groups, followed by Fibrobacterota and Pseudomonadota (Figure 1D). At the genus level, dominant taxa in both groups were *Xylanibacter* and *Rikenellaceae RC9* gut group, followed by *Christensenellaceae R-7* group, *Succiniclasticum*, and *Fibrobacter* (Figure 1E).

**Figure 2:**
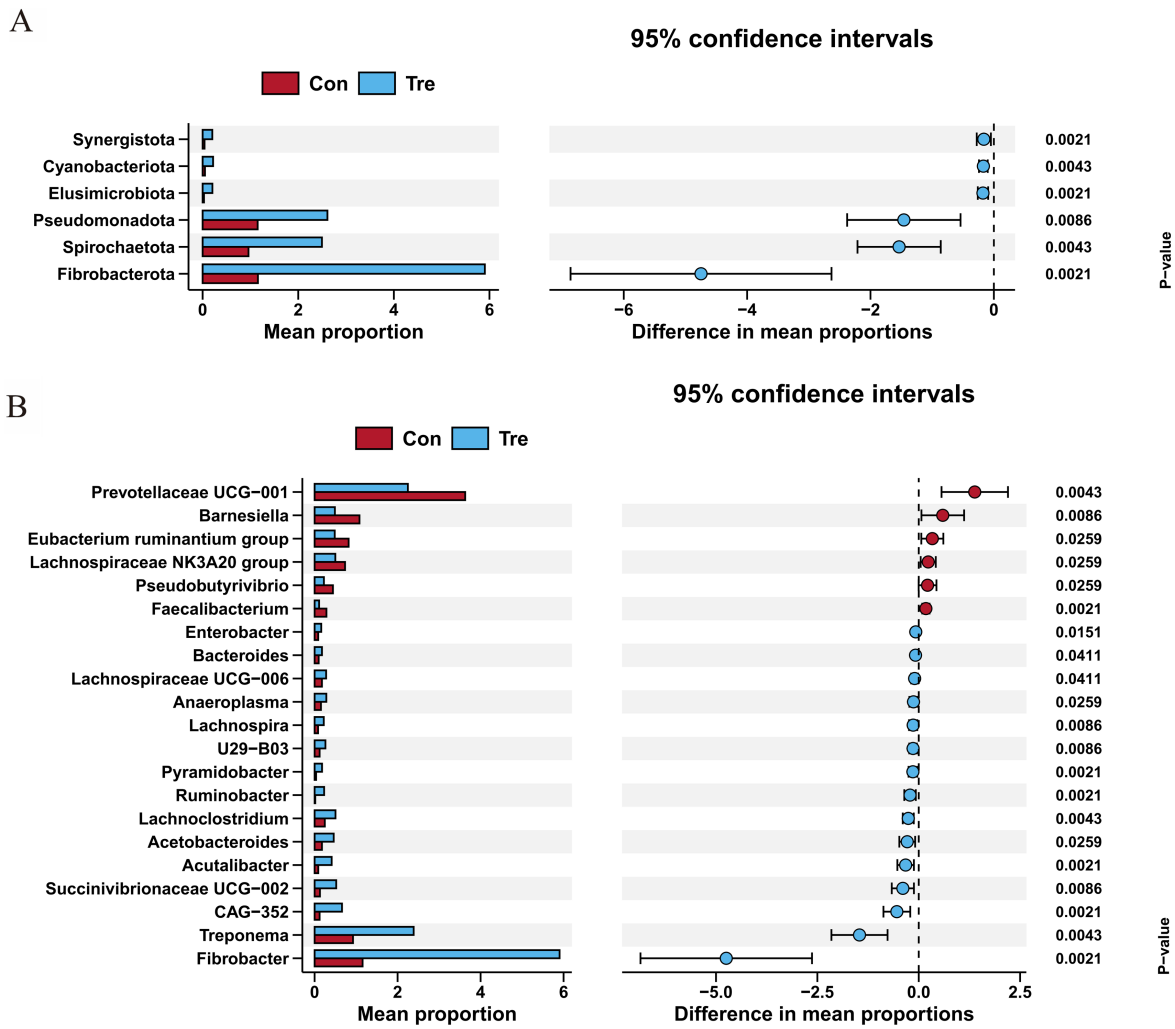
Differences analysis of rumen microbiota of Xinjiang Brown Cattle receiving a diet with cotton straw and apple pomace mixed ensilage. **(A)** Comparison of flora differences at the phylum level. **(B)** Comparison of flora differences at the genus level. a-b means within a row with different subscripts differ when *p* < 0.05.

Wilcoxon test analysis comparisons at phylum and genus levels revealed that the Tre group had significantly higher relative abundances of Cyanobacteriota, Fibrobacterota, Pseudomonadota, Spirochaetota and Synergistota than the Con group (Figure 2A, *p* < 0.05). At the genus level, the relative abundance of *Fibrobacter* was significantly increased and *Prevotellaceae UCG–001* was significantly decreased in the Tre group compared with the Con group (Figure 2B, *p* < 0.05).

**Figure 3:**
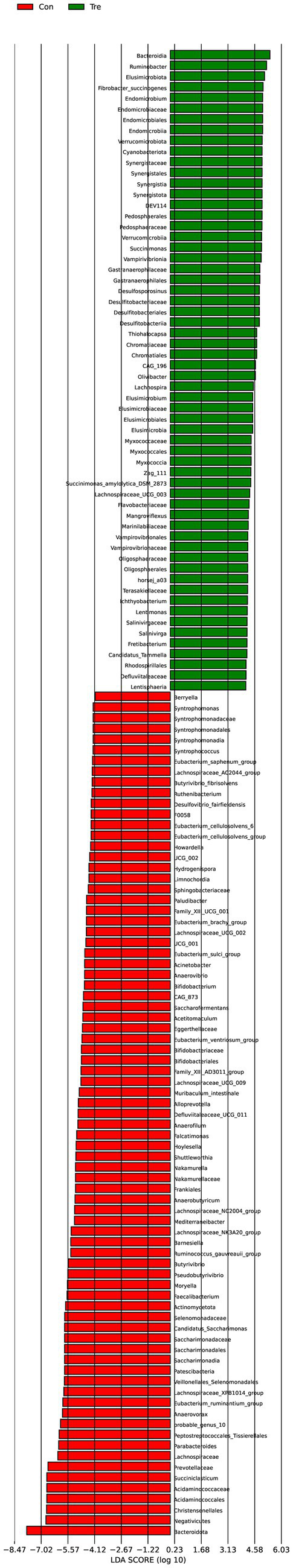
LDA score analysis (LDA ≥ 4).

Linear discriminant analysis (LDA, LDA ≥ 4) highlighted distinct marker taxa for each group. In the Tre group, marker taxa mainly included Bacteroidia, Ruminobacter, Eubacterium, *Fibrobacter Succinogenes*, Endomicrobiales, and Verrucomicrobiota. In contrast, the Con group was characterized by taxa such as Escherichia-Shigella, Enterobacteriaceae, Clostridium, Proteus, Serratia, Pseudomonas, and Acinetobacter (Figure 3).

### 3.5 Effects of different diets on rumen metabolome

**Figure 4:**
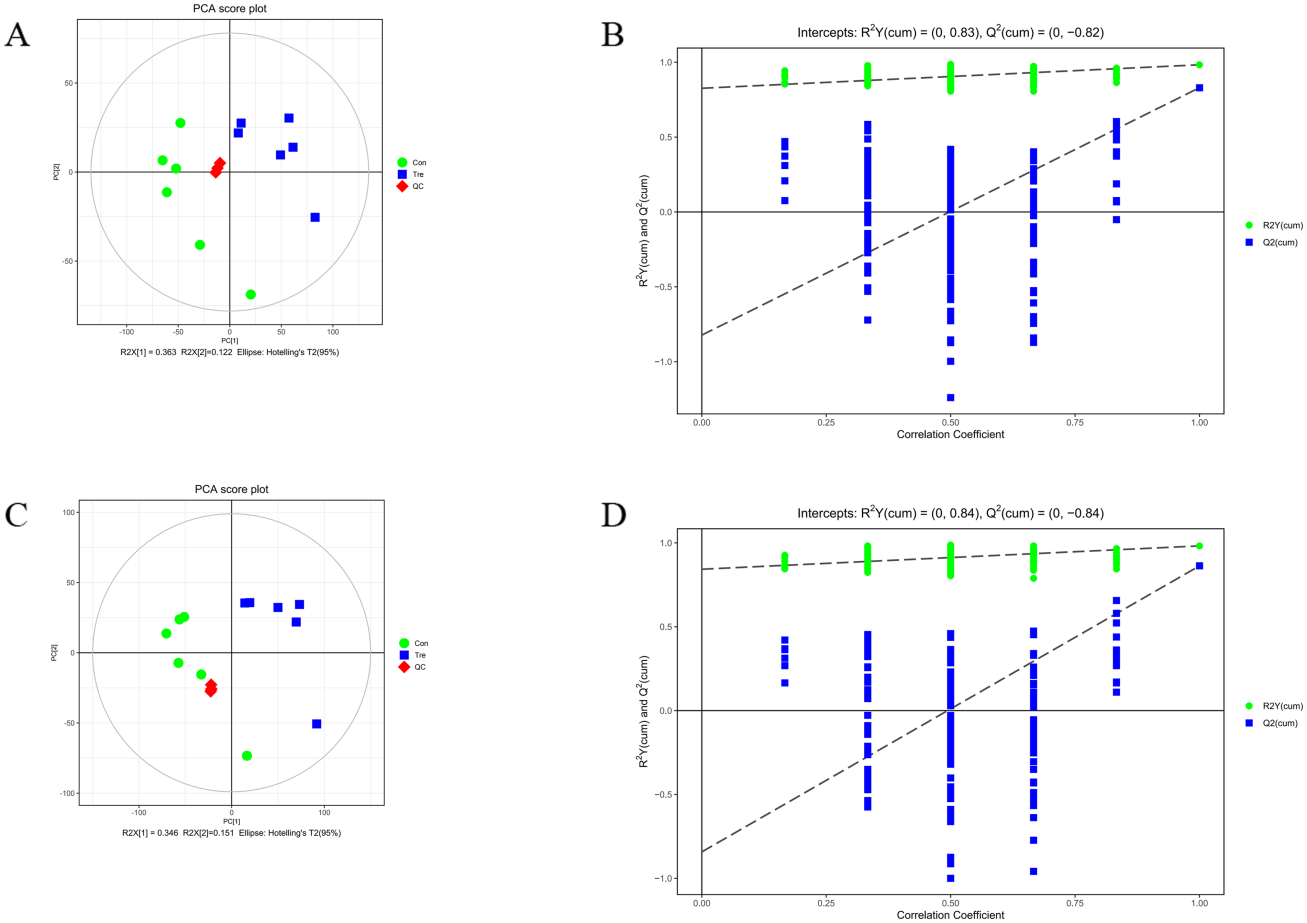
Multivariate statistical analysis of metabolomic data. **(A)** Principal component analysis (PCA) score plot of the control group (Con, green circles) and treatment group (Tre, blue squares) in positive ion mode, with quality control samples (QC) indicated by red diamonds. **(B)** Model validation plot in positive ion mode, showing the changes of cumulative *R*^2^*Y* and *Q*^2^ values with correlation coefficients. **(C)** Principal component analysis (PCA) score plot of the control group (Con, green circles) and treatment group (Tre, blue squares) in negative ion mode, with quality control samples (QC) indicated by red diamonds. **(D)** Model validation plot in negative ion mode, showing the changes of cumulative *R*^2^*Y* and *Q*^2^ values with correlation coefficients.

**Figure 5:**
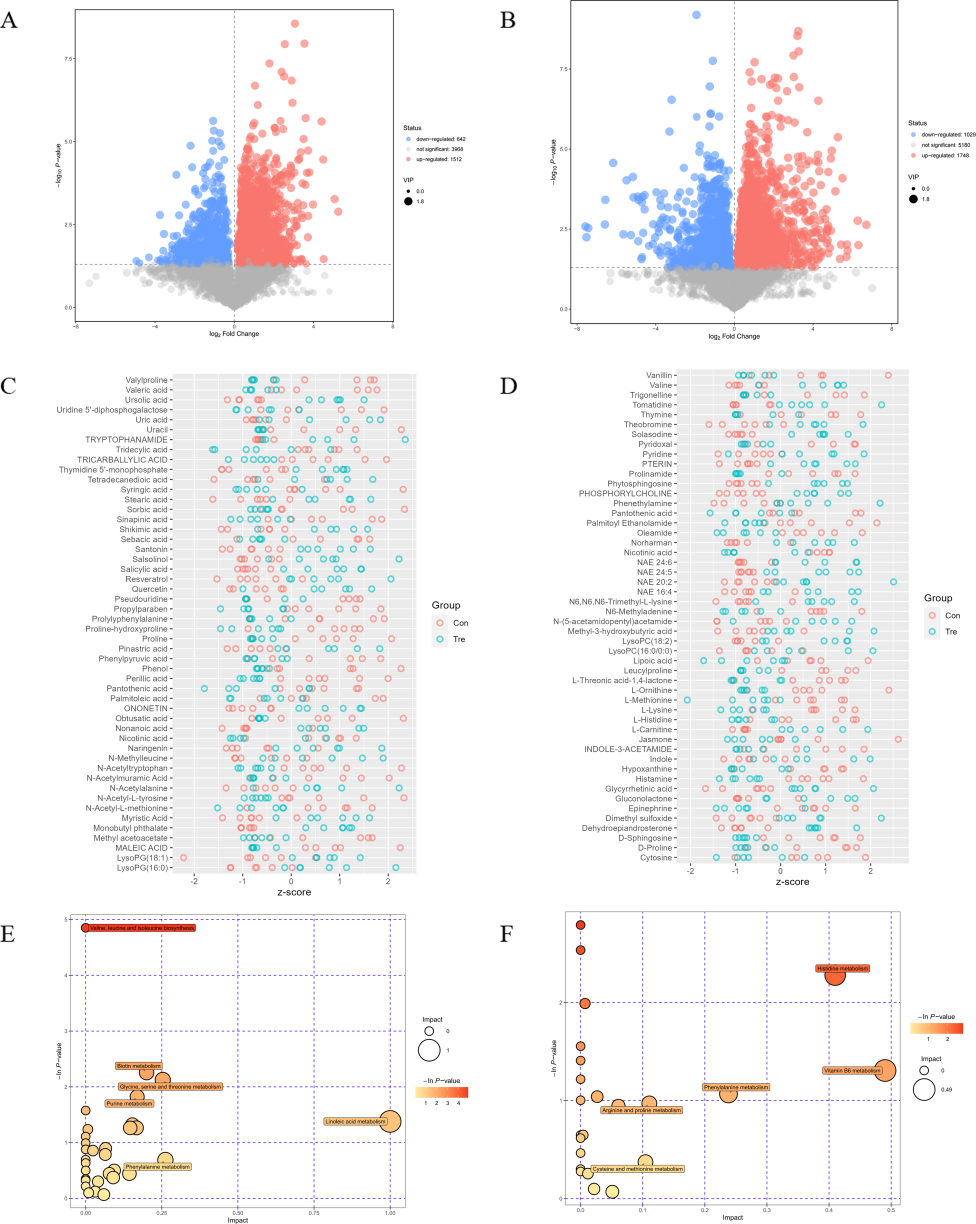
Identification of differential metabolites and KEGG enrichment analysis. **(A)** Volcano plot analysis in positive ion mode. **(B)** Volcano plot analysis in negative ion mode. **(C)** *Z*-score heatmap of significantly changed metabolites between Con group and Tre group in positive ion mode. **(D)** *Z*-score heatmap of significantly changed metabolites between Con group and Tre group in negative ion mode. **(E)** KEGG enrichment plot in positive ion mode. **(F)** KEGG enrichment plot in negative ion mode.

PCA score plot in both positive and negative ion modes showed that Con and Tre samples were distributed in separate regions, indicating diet-driven changes in rumen metabolite profiles (Figures 4A,C). The results of the OPLS-DA model permutation test are shown in (Figures 5B, D). R²Y represents the interpretation rate of the constructed model, while Q² indicates the predictive ability of the model. In theory, the closer the R²Y and Q² values are to 1, the better the model performs; lower values indicate poorer model fit and accuracy. Generally, R²Y and Q² values above 0.5 are considered good, and values above 0.4 are acceptable, indicating that the model is robust and free from overfitting.

In positive ion mode, 2,154 differentially expressed metabolites were identified: 1,512 upregulated and 642 downregulated in Tre vs. Con (*p* < 0.05) (Figure 5A). In negative ion mode, 2,777 differentially expressed metabolites were detected: 1,748 upregulated and 1,029 downregulated (*p* < 0.05) (Figure 5B). *Z*-score plots illustrated specific changes: in positive ion mode, Resveratrol, ursolic acid, uric acid and lysoPG(18:1) were higher in Tre, while valeric acid were lower (Figure 5C). In negative ion mode, N-(5-acetamidopentyl)acetamide and gluconolactone increased in Tre, whereas D-Proline, D-Sphingosine, epinephrine, L-Methionine, L-Threonic acid-1,4-lactone, cytosine, leucylproline and lipoic acid decreased (Figure 5D).

KEGG pathway enrichment analysis showed that in positive ion mode, differentially expressed metabolites were enriched in valine, leucine and isoleucine biosynthesis; linoleic acid metabolism; biotin metabolism; glycine, serine and threonine metabolism; and purine metabolism (*p* < 0.05, Figure 5E). In negative ion mode, enriched pathways included histidine metabolism; vitamin B6 metabolism; phenylalanine metabolism; arginine and proline metabolism; and cysteine and methionine metabolism (*p* < 0.05, Figure 5F).

### 3.6 Correlation analysis among phenotypic traits, rumen bacteria, and metabolites

**Figure 6:**
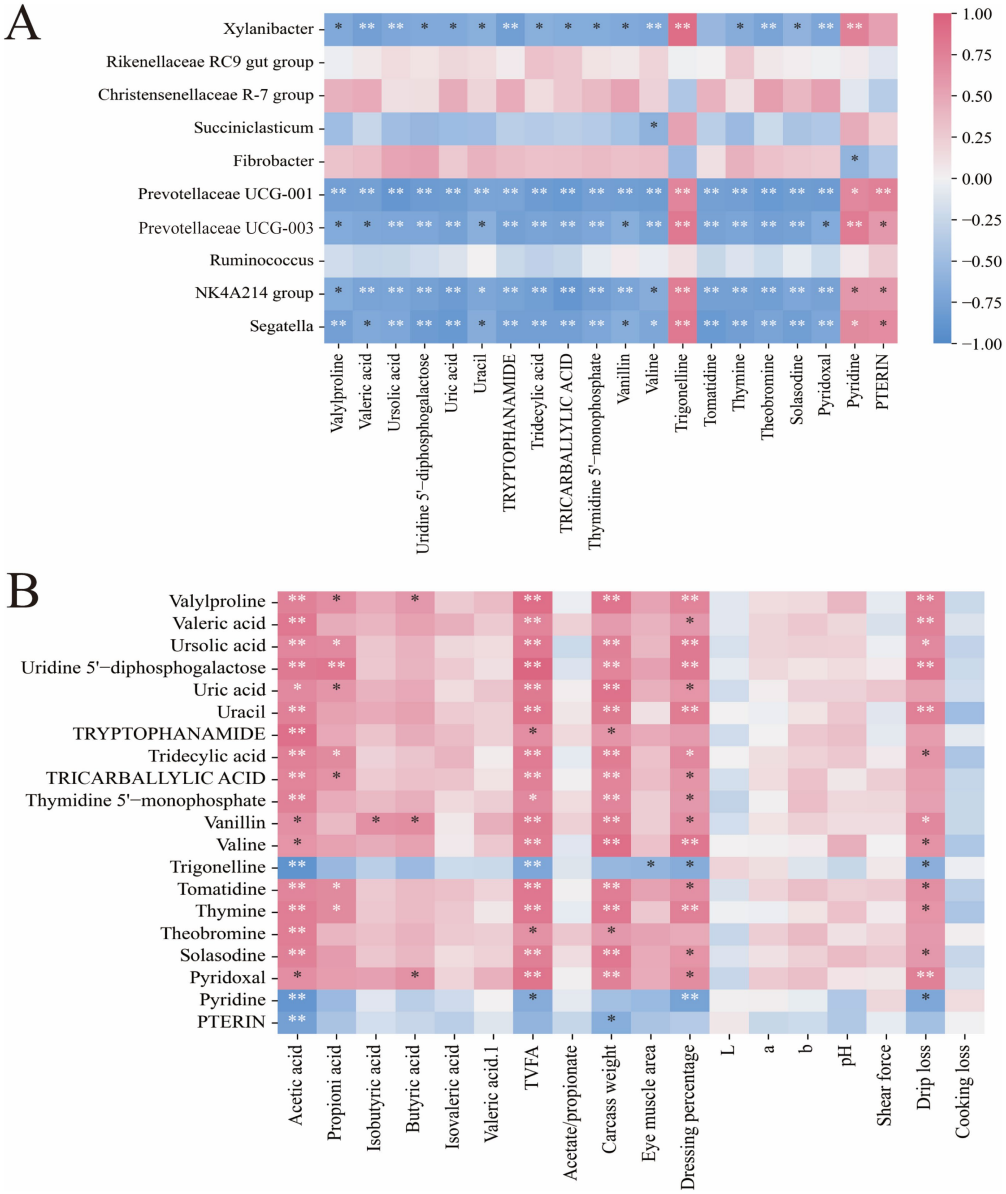
Correlation analysis. **(A)** Association analysis of rumen microbiota and metabolites. **(B)** Association analysis of rumen fluid metabolites and phenotypic traits.

The correlation analysis between rumen microbiota and metabolites revealed that *Prevotellaceae UCG-001* was significantly negatively correlated with valeric acid, valylproline, and ursolic acid, but significantly positively correlated with pterin (*p* < 0.01, Figure 6A). *Fibrobacter* showed a significant negative correlation with pyridine (*p* < 0.05, Figure 6A), whereas *Xylanibacter* was significantly positively correlated with trigonelline and pyridine (*p* < 0.01, Figure 6A). The association analysis between rumen fluid metabolites and phenotypic traits revealed that acetic acid was significantly positively correlated with valeric acid, but significantly negatively correlated with pyridine and pterin (*p* < 0.01, Figure 6B). Carcass weight showed significant positive correlations with metabolites including vanillin, valine, thymine, and tricarballylic acid (*p* < 0.01, Figure 6B). Furthermore, drip loss was significantly positively correlated with valylproline, uridine 5′-diphosphogalactose, and uracil (*p* < 0.01, Figure 6B).

## 4 Discussion

Roughage serves as a core component of ruminant diets, with its quality directly influencing production efficiency and cattle growth performance. High-quality roughage not only enhances feed conversion efficiency but also optimizes nutrient supply, thereby significantly promoting the growth and production performance of ruminants (22). Previous studies have shown that cotton by-products can serve as effective sources of fiber, fat, and protein in feedlot diets, and that apple pomace contains high levels of digestible neutral detergent fiber (NDF) (23, 24). Warner et al. found that including cotton by-products in late-stage finishing diets did not adversely affect the performance or carcass characteristics of beef cattle (25). Danish et al. investigated the effects of incorporating varying levels of apple pomace into the diets of crossbred calves and reported that its inclusion had no significant impact on dry matter intake, growth performance, or feed conversion ratio (26). In this study, cotton straw and apple pomace were combined and fed to Xinjiang brown cattle. The results showed that this dietary treatment had no significant effect on the ADG of the animals, but it significantly reduced carcass weight, dressing percentage, and the drip loss of the longissimus dorsi muscle. These results indicate that, compared to feeding cotton straw or apple pomace alone, their combined feeding exerted a detrimental effect on the slaughter performance of Xinjiang Brown Cattle. This adverse outcome may be attributed to alterations in fermentation patterns and energy utilization efficiency induced by the mixed diet, which subsequently inhibited effective muscle deposition, ultimately leading to reduced carcass yield.

Rumen fermentation parameters, including pH, NH_3_-N, MCP, and VFA, indicate rumen stability and microbial activity. Rumen pH in both groups remained within the normal 5.5–7.0 range (27, 28), indicating stable fermentation. Volatile fatty acids (VFA) are the primary end-products of ruminal fermentation, mainly comprising acetate, propionate, and butyrate, and serve as the principal energy source for ruminants (29). The present study found that the mixed feeding of cotton straw and apple pomace significantly reduced the concentrations of total volatile fatty acids (TVFA), acetic acid, and propionic acid in the rumen. Acetate is primarily produced by fibrolytic bacteria through the fermentation of structural carbohydrates (e.g., cellulose and hemicellulose), whereas propionate is a key precursor for gluconeogenesis in ruminants and plays a critical role in maintaining blood glucose homeostasis and energy supply (30, 31). Therefore, it is speculated that the mixed feeding of cotton straw and apple pomace reduced the ruminal fermentation capacity in Xinjiang Brown cattle, potentially due to the high lignification degree of cotton straw limiting fiber degradation, thereby preventing the positive effects of apple pomace from being fully realized.

The rumen hosts a complex microbial ecosystem, containing bacteria, protozoa, fungi, archaea (32). Diet markedly affects community and fermentation mode (32). Bacteroidetes support carbohydrate digestion (33), while Bacillota include various fiber-degrading genera (34). Consistent with Zened et al. (35), the dominant phyla (Bacteroidetes and Bacillota) remained unchanged with dietary changes. According to the LEfSe analysis results, Bacteroidia, Ruminobacter, Elusimicrobiota, *Fibrobacter succinogenes*, and *Endomicrobium* were the top five significantly enriched microbial taxa with the highest LDA scores in the Tre group. Among these, Bacteroidia, as a core bacterial group in the rumen, is primarily involved in the degradation of hemicellulose and the production of VFA (36–38). Endomicrobium, belonging to Elusimicrobia, is an obligate anaerobic intracellular symbiotic bacterium that participates in lignocellulose degradation mainly by secreting cellulase and hemicellulase (39). *Fibrobacter succinogenes*, a highly efficient cellulolytic bacteria, attaches to plant cell walls and secretes cellulases and hemicellulases to degrade fiber (40–42). In the Tre group, the significant enrichment of the aforementioned taxa may be associated with the higher cellulose and lignin content in the diet of this group. This shift in microbial community structure reflects the adaptive changes of the rumen microbiota to an increased fiber substrate load. However, despite the increased relative abundance of certain fiber-degrading bacteria, the TVFA concentration in the Tre group exhibited a decline. This discrepancy may be attributed to the complex interaction networks among rumen microorganisms, as well as the dynamic balance between substrate availability and fermentation efficiency. In contrast, Syntrophomonadales and its subordinate taxa (including Syntrophomonadaceae, Syntrophomonas, and Berryella) were significantly enriched in the Con group. This taxon is a key functional group in the rumen involved in the degradation of long-chain fatty acids (LCFA), primarily converting LCFA into acetate through the *β*-oxidation pathway (43). Due to the lower lignin content in the diet of the Con group, the degradability of its fiber components may be higher, thereby providing more favorable conditions for the synthesis of short-chain fatty acids.

Metabolomics helps reveal how microbial communities contribute to digestion, absorption, and energy utilization in ruminants and elucidates host–microbe interactions. LC–MS metabolomics revealed substantial differences in rumen metabolites between groups, supported by PLS-DA and permutation tests, indicating the Tre diet significantly altered rumen metabolism. Increases in N-Acetyl-L-methionine (associated with improved feed efficiency and enhanced oxidative stability in meat) and N-Acetyl-L-tyrosine (a tyrosine source important in growth) suggest potential benefits of the Tre diet for feed efficiency and meat quality (44–46). Resveratrol possesses antioxidant, antibacterial, anti-inflammatory, and metabolic regulatory properties (47). Studies have indicated that dietary resveratrol exerts therapeutic effects on oxidative stress and inflammation induced by early weaning (48, 49), heat stress (50), mycotoxins (51), and bacterial diseases, and is beneficial for animal growth, development, and product quality (52). Quercetin, a natural flavonoid compound widely present throughout the plant kingdom, exhibits diverse pharmacological activities and can serve as a safe dietary polyphenol to inhibit lipid oxidation (53). Therefore, the elevated concentrations of quercetin and resveratrol likely originate primarily from the apple pomace included in the diet, thereby contributing to enhanced immune function and antioxidant capacity in Xinjiang Brown Cattle.

Rumen microbiota and their metabolites not only influence the growth and development of the host but also collectively shape the host’s health status through microbe-metabolite interactions (54). The level of ursolic acid was significantly increased in the Tre group, which may be directly attributed to the supplementation of apple pomace in the Tre group diet. Previous studies have shown that ursolic acid possesses significant bioactivities, including anti-inflammatory, antioxidant, and lipid metabolism-regulating effects (55, 56). In addition, ursolic acid has been shown to improve host immune status by modulating the intestinal microbiota (57, 58). In this study, the concentration of ursolic acid was negatively correlated with the relative abundances of the potential pathogenic bacteria *Prevotellaceae UCG-001* and *Prevotellaceae UCG-003* (59). Therefore, we hypothesize that ursolic acid may improve the composition of the intestinal microbiota, such as by inhibiting pathogenic bacteria, thereby enhancing the immune status of Xinjiang Brown cattle. However, this hypothesis requires further validation. Tridecylic acid, an odd-chain fatty acid, is commonly used as a reliable indicator for assessing ruminal microbial biomass and fermentation activity (60). Correlation analysis further revealed that tridecylic acid was significantly negatively correlated with Segatella, the NK4A214 group, Ruminococcus, and Xylanibacter. This coordinated variation between the metabolite and the microbiota explains the reduction in T-VFA production in the Tre group, suggesting that the mixed silage of cotton straw and apple pomace may modulate ruminal fermentation patterns by reshaping the microbial community structure. Furthermore, the metabolite trigonelline was significantly negatively correlated with drip loss, indicating its potential to improve meat water-holding capacity and tenderness. Therefore, these findings support the notion that microbe-metabolite interactions regulate nutrient digestion and absorption, thereby influencing host growth performance.

## 5 Conclusion

Complete replacement of corn silage with a fermented cotton straw-apple pomace mixture adversely affected the slaughter parameters of Xinjiang Brown Cattle, while improving meat quality to some extent. Specifically, compared with the Con group, feeding the fermented mixture resulted in significantly lower carcass weight, as well as reduced ruminal VFA concentrations. However, meat water-holding capacity was significantly improved in the Tre group. In addition, this dietary substitution induced significant alterations in the ruminal bacterial community structure and metabolic profiles. In conclusion, the findings of this study indicate that the fermented cotton straw-apple pomace mixture is currently not suitable as a complete substitute for corn silage; future research should focus on further optimizing fermentation strategies to enhance its nutritional value and feeding efficacy.

## Data Availability

The raw sequencing data of rumen bacteria have been deposited in the NCBI with the accession number PRJNA1397473.
